# Supplementary material for: Vampire bats in Belize harbor multiple Trypanosoma cruzi genotypes: Implications for parasite transmission at the wildlife–domestic–human interface
Source: PLoS Negl Trop Dis. 2026 Sep 11;20(9):e0013852. doi: 10.1371/journal.pntd.0013852 (PMC13592685; doi:10.1371/journal.pntd.0013852)
Supplement: Supplementary file 1 [file pntd.0013852.s001.docx]

**Supplemental Table 1.** ANOVA table for GLMM 1 (individual-level predictors) of *T. cruzi* infection (*n* = 207). The random intercept of bat identity was estimated as σ^2^ = 0.41.

| **Predictor** | ***χ^2^*** | **df** | ***p*** |
| --- | --- | --- | --- |
| Age | 0.01 | 1 | 0.91 |
| Sex | 1.72 | 1 | 0.19 |
| Reproductive status | 0.50 | 1 | 0.48 |
| Age * sex | 2.09 | 1 | 0.15 |
| Sex * reproductive status | 0.47 | 1 | 0.49 |
