# Supplementary material for: Vampire bats in Belize harbor multiple Trypanosoma cruzi genotypes: Implications for parasite transmission at the wildlife–domestic–human interface
Source: PLoS Negl Trop Dis. 2026 Sep 11;20(9):e0013852. doi: 10.1371/journal.pntd.0013852 (PMC13592685; doi:10.1371/journal.pntd.0013852)
Supplement: Supplementary file 2 [file pntd.0013852.s002.docx]

**Supplemental Table 2**. Results for GLMM 1 (individual-level predictors) of *T. cruzi* infection (*n* = 207). Reference levels include juveniles and subadults, females, and non-reproductive bats. Results are presented without interactions to improve interpretation of the main effects.

| **Predictor** | **β** | **SE** | **OR** | **95% CI** | | ***p*** |
| --- | --- | --- | --- | --- | --- | --- |
| Intercept | -0.37 | 0.39 | – | – | – | 0.35 |
| Adult | -0.05 | 0.40 | 0.96 | 0.43 | 2.10 | 0.91 |
| Male | 0.45 | 0.33 | 1.56 | 0.81 | 3.01 | 0.18 |
| Reproductive | -0.25 | 0.37 | 0.78 | 0.38 | 1.60 | 0.49 |
