# Supplementary material for: Vampire bats in Belize harbor multiple Trypanosoma cruzi genotypes: Implications for parasite transmission at the wildlife–domestic–human interface
Source: PLoS Negl Trop Dis. 2026 Sep 11;20(9):e0013852. doi: 10.1371/journal.pntd.0013852 (PMC13592685; doi:10.1371/journal.pntd.0013852)
Supplement: Supplementary file 3 [file pntd.0013852.s003.docx]

**Supplemental Table 3.** ANOVA table for GLMM 2 (spatiotemporal predictors) of *T. cruzi* infection (*n* = 193). The random intercept of bat identity was estimated as σ^2^ = 0.33.

| **Predictor** | ***χ^2^*** | **df** | ***p*** |
| --- | --- | --- | --- |
| Age | 0.02 | 1 | 0.88 |
| Sex | 0.91 | 1 | 0.34 |
| Reproductive status | 0.13 | 1 | 0.72 |
| Site | 4.81 | 1 | 0.03 |
| Year | 9.88 | 1 | 0.002 |
| Site * year | 0.09 | 1 | 0.76 |
