# Supplementary material for: Vampire bats in Belize harbor multiple Trypanosoma cruzi genotypes: Implications for parasite transmission at the wildlife–domestic–human interface
Source: PLoS Negl Trop Dis. 2026 Sep 11;20(9):e0013852. doi: 10.1371/journal.pntd.0013852 (PMC13592685; doi:10.1371/journal.pntd.0013852)
Supplement: Supplementary file 4 [file pntd.0013852.s004.docx]

**Supplemental Table 4**. Results for GLMM 2 (spatiotemporal predictors) of *T. cruzi* infection (*n* = 193). Reference levels include juveniles and subadults, females, non-reproductive bats, LAR, and 2019. Results are presented without the site*year interaction to improve interpretation of main effects of site and year.

| **Predictor** | **β** | **SE** | **OR** | **95% CI** | | ***p*** |
| --- | --- | --- | --- | --- | --- | --- |
| Intercept | -0.52 | 0.41 | – | – | – | 0.21 |
| Adult | -0.06 | 0.43 | 0.94 | 0.41 | 2.20 | 0.89 |
| Male | 0.35 | 0.36 | 1.42 | 0.70 | 2.87 | 0.33 |
| Reproductive | -0.15 | 0.40 | 0.86 | 0.40 | 1.87 | 0.70 |
| KK | -1.07 | 0.48 | 0.34 | 0.13 | 0.89 | 0.03 |
| 2022 | 1.28 | 0.41 | 3.59 | 1.61 | 7.99 | 0.002 |
