# Supplementary material for: Vampire bats in Belize harbor multiple Trypanosoma cruzi genotypes: Implications for parasite transmission at the wildlife–domestic–human interface
Source: PLoS Negl Trop Dis. 2026 Sep 11;20(9):e0013852. doi: 10.1371/journal.pntd.0013852 (PMC13592685; doi:10.1371/journal.pntd.0013852)
Supplement: Supplementary file 5 [file pntd.0013852.s005.docx]

**Supplemental Table 5.** ANOVA table for GLMM 3 (temporal predictors) of *T. cruzi* infection (*n* = 169). The random intercept of bat identity was estimated as σ^2^ = 0.57.

| **Predictor** | ***χ^2^*** | **df** | ***p*** |
| --- | --- | --- | --- |
| Age | 0.16 | 1 | 0.69 |
| Sex | 1.07 | 1 | 0.30 |
| Reproductive status | 1.08 | 1 | 0.30 |
| Year | 9.89 | 1 | 0.007 |
