# Supplementary material for: Vampire bats in Belize harbor multiple Trypanosoma cruzi genotypes: Implications for parasite transmission at the wildlife–domestic–human interface
Source: PLoS Negl Trop Dis. 2026 Sep 11;20(9):e0013852. doi: 10.1371/journal.pntd.0013852 (PMC13592685; doi:10.1371/journal.pntd.0013852)
Supplement: Supplementary file 6 [file pntd.0013852.s006.docx]

**Supplemental Table 6.** Results for GLMM 3 (temporal predictors) of *T. cruzi* infection (*n* = 169). Reference levels include juveniles and subadults, females, non-reproductive bats, and 2019.

| **Predictor** | **β** | **SE** | **OR** | **95% CI** | | ***p*** |
| --- | --- | --- | --- | --- | --- | --- |
| Intercept | -0.56 | 0.45 | – | – | – | 0.21 |
| Adult | 0.18 | 0.46 | 1.20 | 0.49 | 2.92 | 0.69 |
| Male | 0.40 | 0.39 | 1.50 | 0.70 | 3.21 | 0.30 |
| Reproductive | -0.43 | 0.42 | 0.65 | 0.29 | 1.47 | 0.30 |
| 2021 | -0.54 | 0.68 | 0.58 | 0.16 | 2.20 | 0.43 |
| 2022 | 1.33 | 0.46 | 3.79 | 1.53 | 9.36 | 0.004 |
