# Supplementary material for: Vampire bats in Belize harbor multiple Trypanosoma cruzi genotypes: Implications for parasite transmission at the wildlife–domestic–human interface
Source: PLoS Negl Trop Dis. 2026 Sep 11;20(9):e0013852. doi: 10.1371/journal.pntd.0013852 (PMC13592685; doi:10.1371/journal.pntd.0013852)
Supplement: Supplementary file 7 [file pntd.0013852.s007.docx]

**Supplemental Table 7.** Haplotype composition and DTU assignment per bat-derived *T. cruzi* sample (LAR site, 2022).

| **Sample ID** | **Site** | **Year** | **Haplotypes** | **DTU** |
| --- | --- | --- | --- | --- |
| Belize_Bat-1009 | LAR | 2022 | H1, H2, H3, H4, H5, H6, H7, H8, H9 | TcBat |
| Belize_Bat-1027 | LAR | 2022 | H1 | TcBat |
| Belize_Bat-1037 | LAR | 2022 | H1 | TcI |
| Belize_Bat-1043.01 | LAR | 2022 | H1 | TcBat |
| Belize_Bat-1718 | LAR | 2022 | H1, H2, H3, H4, H5, H6, H7, H8, H9 | TcBat |
| Belize_Bat-79.01 | LAR | 2022 | H1, H2, H3, H4 | TcBat |
| Belize_Bat-1743 | LAR | 2022 | H1 | TcIV |
| Belize_Bat-1768 | LAR | 2022 | H1, H2, H3, H4, H5, H6, H7, H8 | TcVI |
| Belize_Bat-1773 | LAR | 2022 | H1 | TcI |
| Belize_Bat-1774 | LAR | 2022 | H1 | TcV |
